# Graphene oxide selectively targets cancer stem cells, across multiple tumor types: Implications for non-toxic cancer treatment, via “differentiation-based nano-therapy”

**DOI:** 10.18632/oncotarget.3348

**Published:** 2015-02-24

**Authors:** Marco Fiorillo, Andrea F. Verre, Maria Iliut, Maria Peiris-Pagés, Bela Ozsvari, Ricardo Gandara, Anna Rita Cappello, Federica Sotgia, Aravind Vijayaraghavan, Michael P. Lisanti

**Affiliations:** ^1^ The Manchester Centre for Cellular Metabolism (MCCM), Institute of Cancer Sciences, University of Manchester, UK; ^2^ The Breakthrough Breast Cancer Research Unit, Institute of Cancer Sciences, University of Manchester, UK; ^3^ The Department of Pharmacy, Health and Nutritional Sciences, The University of Calabria, Italy; ^4^ School of Materials and National Graphene Institute, University of Manchester, UK

**Keywords:** nanomaterials, graphene oxide, cancer stem cells, multiple cancer types: breast, ovarian

## Abstract

Tumor-initiating cells (TICs), a.k.a. cancer stem cells (CSCs), are difficult to eradicate with conventional approaches to cancer treatment, such as chemo-therapy and radiation. As a consequence, the survival of residual CSCs is thought to drive the onset of tumor recurrence, distant metastasis, and drug-resistance, which is a significant clinical problem for the effective treatment of cancer. Thus, novel approaches to cancer therapy are needed urgently, to address this clinical need. Towards this end, here we have investigated the therapeutic potential of graphene oxide to target cancer stem cells. Graphene and its derivatives are well-known, relatively inert and potentially non-toxic nano-materials that form stable dispersions in a variety of solvents. Here, we show that graphene oxide (of both big and small flake sizes) can be used to selectively inhibit the proliferative expansion of cancer stem cells, across multiple tumor types. For this purpose, we employed the tumor-sphere assay, which functionally measures the clonal expansion of single cancer stem cells under anchorage-independent conditions. More specifically, we show that graphene oxide effectively inhibits tumor-sphere formation in multiple cell lines, across 6 different cancer types, including breast, ovarian, prostate, lung and pancreatic cancers, as well as glioblastoma (brain). In striking contrast, graphene oxide is non-toxic for “bulk” cancer cells (non-stem) and normal fibroblasts. Mechanistically, we present evidence that GO exerts its striking effects on CSCs by inhibiting several key signal transduction pathways (WNT, Notch and STAT-signaling) and thereby inducing CSC differentiation. Thus, graphene oxide may be an effective non-toxic therapeutic strategy for the eradication of cancer stem cells, via differentiation-based nano-therapy.

## INTRODUCTION

Cancer stem cells (CSCs) are resistant to conventional therapeutic approaches [1-3]. As a consequence, they have been directly implicated in the disease pathogenesis of tumor recurrence and distant metastasis [4, 5]. In addition, drug-resistant CSCs have been linked to unfavorable clinical outcomes, across different tumor types [6-8]. As only a very small percentage of cancer cells have “stem-like” and “tumor-initiating” properties, they are difficult to study and their key distinguishing features remain relatively uncharacterized, although they appears to resist both chemo-therapy and radiation.

Interestingly, CSCs share many properties with normal stem cells, including immortality and resistance to stress, as well as asymmetric cell division [9, 10]. A particular distinguishing characteristic of CSCs is their ability to initiate tumors and to undergo anchorage-independent growth, when cultured in suspension [11]. Under these particular cell culture conditions, CSCs proliferate and form 3D-spheroid-like structures, containing CSCs and progenitor cells, which are known as “tumor-spheres” or “onco-spheres” [12, 13]. In striking contrast, the vast majority of non-CSCs undergo a specialized form of apoptosis in suspension cultures, called anoikis. Importantly, each 3D-spheroid originates from the clonal proliferation of a single CSC, is not to due to the self-aggregation of cancer cells. Thus, tumor-sphere formation is an efficient means to selectively enrich for CSCs. The CSC population is resistant to DNA-damage, and shows lower levels of ROS production, as well [14, 15]. 3D-tumor-spheres derived from breast cancer cells are also known as mammo-spheres [12, 13].

Clinically, there is an urgent need to identify new therapeutic strategies for selectively targeting CSCs. Here, we show that graphene oxide (GO) demonstrates this selectivity. As such, our current study provides a new rationale for exploiting graphene oxide itself as an anti-cancer therapeutic, rather than simply as a drug-delivery agent [16].

## RESULTS

### GO flakes target breast cancer stem cells

Here, we tested the efficacy of graphene oxide as a potential new anti-cancer agent, for the selective targeting of CSCs. Graphene, described as two-dimensional sheets of carbon atoms without any additional functional groups, does not forms stable dispersions in water or other biologically relevant solvents [17]. On the other hand, graphene oxide [18] is the water-soluble derivative of graphene which can be produced with various sizes and bearing varied functional groups and which can be more easily manipulated experimentally, especially in biological systems. Sterile GO dispersions were prepared in a 5% mixture of DMSO in double-distilled water for these studies.

**Figure 1:**
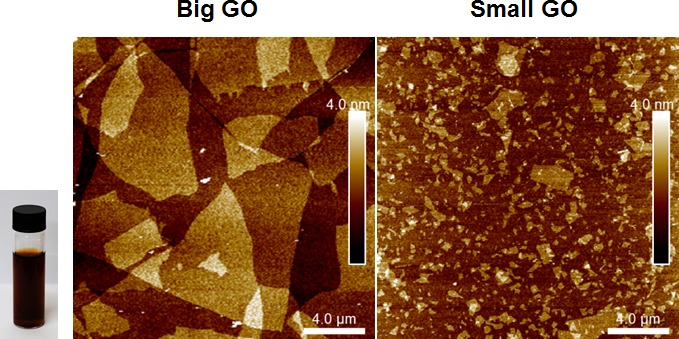
Graphene oxide (GO) grades Left and Right panels show atomic force microscopy images of graphene oxide on a silicon dioxide substrate indicating the flake size distribution and monolayer thickness of the flakes. Inset shows a vial of b-GO stock dispersion in DMSO and water, at concentration of 2.3 mg/ml.

**Figure 2:**
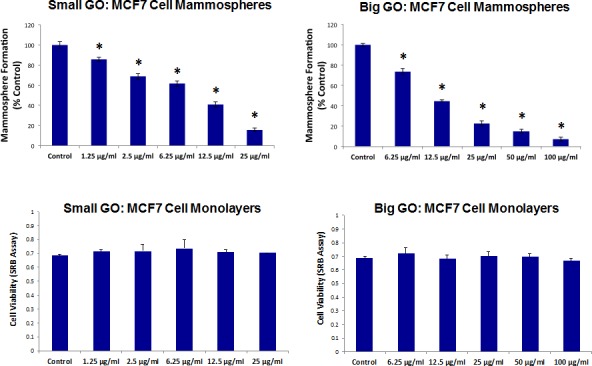
Graphene oxide (GO) selectively targets cancer stem cells (CSCs) in breast cancer cells Upper Panels. Note that GO (big and small flakes) inhibits the anchorage-independent proliferation of MCF7 CSCs, as evidenced by inhibition of mammosphere formation. Lower Panels. In contrast, GO (big and small flakes) does not affect cell viability of the total MCF7 cell population. An * indicates p < 0.05 (Student's t-test).

Initially, we tested the ability of GO to affect CSC proliferation, using MCF7 cells, a well-established ER(+) breast cancer cell line. Two grades of GO were used, small GO (s-GO) with flake sizes of 0.2–2 μm and big GO (b-GO) with flake sizes of 5–20 μm, to represent two size classes, where the flakes are either smaller or larger than the target cells (Figure 1). For this purpose, we assessed the effects of graphene oxide on the anchorage-independent clonal expansion of MCF7 CSCs, using the tumor-sphere assay. This functional assay directly measures CSCs proliferative expansion [12]. Figure 2 shows the results of this analysis. Using s-GO flakes, we observed a dose-dependent inhibition of tumor-sphere formation, in the range of 1.25 to 25 μg/ml, with an IC-50 of ~12.5 μg/ml. Similarly, using b-GO flakes, we also observed a dose-dependent inhibition of tumor-sphere formation, in the range of 6.25 to 100 μg/ml, again with an IC-50 of ~12.5 μg/ml. Importantly, both small and big GO flakes did not affect the viability of the bulk non-CSC population of MCF7 cells, indicating selectivity towards CSCs (Figure 2).

### GO flakes target CSCs, across multiple cancer types

Since both the small and big GO flakes showed similar potency, we focused more on evaluating the efficacy of b-GO flakes. We next evaluated whether GO also showed efficacy against CSCs from multiple cancer types, such as ovarian, prostate, pancreatic and lung cancers, as well as glioblastoma (brain) (the six cell lines tested are summarized in Table 1). For simplicity, we tested b-GO flakes at two doses, namely 25 and 50 μg/ml.

**Table 1:** Six Cancer Cell Models With Board Applicability

Cancer Types	Cell Lines
**Breast(ER+)**	MCF7
**Ovarian**	SKQV3
**Prostate**	PC3
**Pancreatic**	MIA-PaCa-2
**Lung**	A549
**Ghoblastoma**	U87MG

**Figure 3:**
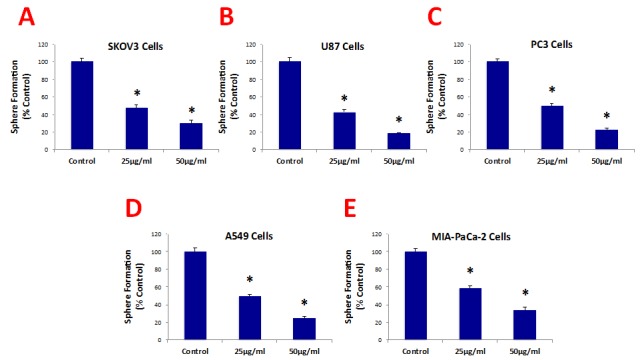
Graphene oxide (GO) selectively targets cancer stem cells (CSCs) of multiple cancer cell types GO (big flakes) inhibits the anchorage-independent proliferation of SKOV3 ovarian cancer cells (A), U87 glioblastoma cells (B), PC3 prostate cancer cells (C), A549 lung cancer cells (D), as well as pancreatic cancer cells (E), in a concentration-independent manner. These results indicate that GO inhibits sphere formations of multiple cancer types. An * indicates p < 0.05 (Student's t-test).

**Figure 4:**
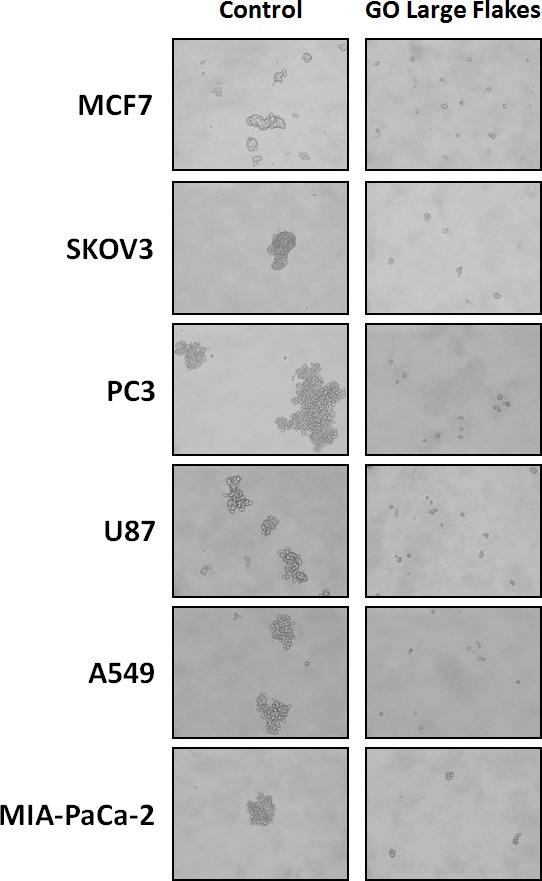
Graphene oxide (GO) selectively targets cancer stem cells (CSCs) of multiple cancer cell types Representative images showing that GO (big flakes, 25μg/ml) inhibits the anchorage-independent proliferation of MCF7 breast cancer cells, SKOV3 ovarian cancer cells, PC3 prostate cancer cells, U87 glioblastoma cells, A549 lung cancer cells as well as MIA-PaCa-2 pancreatic cancer cells.

**Figure 5:**
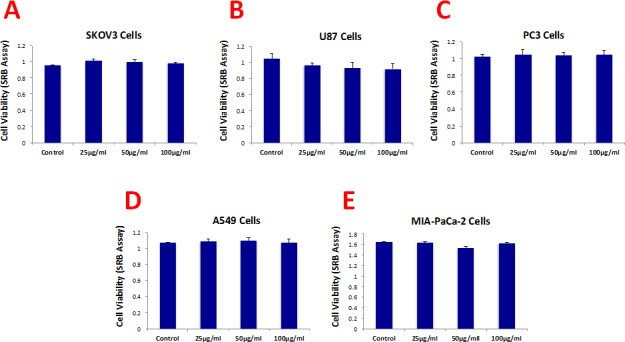
Graphene oxide (GO) does not affect cell viability of the total population of cancer cells Cell viability was assessed using an SRB assay. Note that GO (big flakes) does not affect cell viability of the total cell population of SKOV3 ovarian cancer cells (A), U87 glioblastoma cells (B), PC3 prostate cancer cells (C), A540 lung cancer cells (D) as well as MIA-PaCa-2 pancreatic cancer cells.

Figure 3 shows that b-GO flakes also effectively inhibited tumor-sphere formation in these 5 other cell lines. Thus, our results indicate that GO must be targeting a relatively specific and highly-conserved phenotypic property of CSCs, across multiple cancer types. Representative images of tumor-sphere inhibition by GO treatment are shown in Figure 4. Interestingly, the viability of bulk non-CSCs from these five cancer cell lines (SKOV3, PC3, A549, Mia PaCa2, and U-87) was not affected by GO (Figure 5), further highlighting its specificity and selectivity for CSCs.

**Figure 6:**
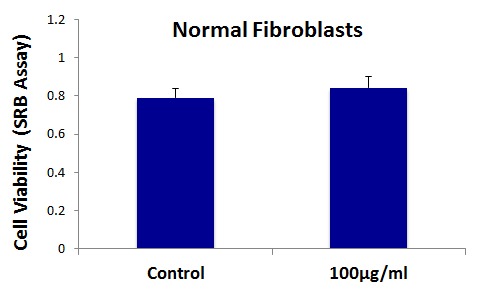
Graphene oxide (GO) does not affect the cell viability normal fibroblasts Cell viability of hTERT-BJ1 fibroblasts was assessed using an SRB assay. Note that GO (big flakes) does not affect cell viability of the total cell population of normal fibroblasts.

Importantly, b-GO flakes also did not affect the viability of a normal skin fibroblast cell line (hTERT-BJ1), indicating that GO is relatively non-toxic for normal body cells (Figure 6). This is consistent with the findings of many other laboratories, i.e., that GO is non-toxic for multiple normal cell types [19, 20].

### GO-based mechanistic studies: Effects on well-established CSC signaling pathways

To gain mechanistic insights into how GO flakes target cancer stem cells, we next analyzed their effects on a series of well-established signal transduction pathways, which have been shown to contribute towards “stemness” [21-23].

**Figure 7:**
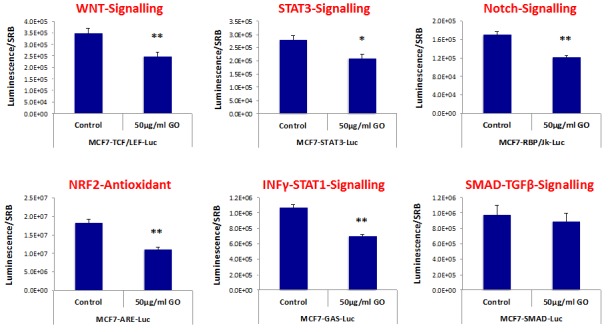
Graphene oxide (GO) inhibits signaling pathways related to cancer stem cells, antioxidant responses and interferon MCF7 breast cancer cells carrying luciferase-reporters (Cignal, QIAGEN) were generated to monitor the activation of a variety of signaling networks, including Wnt, STAT3, Notch, NRF2-dependent antioxidant responses, Interferonγ-STAT1 and SMAD-TGFβ pathways. MCF7-Luc reporter cells were treated with GO (big flakes) for 48 hours and luminescence was determined as a measure of pathway activation status. Note that GO inhibits cancer stem cell signaling (WNT, STAT3 and Notch), NRF2-antioxidant responses, as well as INFΥ-STAT1 signaling. No effects were observed for the SMAD-TGFβ-pathway. An * indicates p < 0.05; ** indicates p < 0.01 (Student's t-test).

For this purpose, we used a panel of MCF7 cell lines that were stably-transfected with different luciferase reporters, that allows one to quantitatively measure the activation-state of a given signal transduction pathway. Interestingly, Figure 7 shows that a number of signaling pathways were significantly inhibited by GO treatment. More specifically, GO treatment inhibited WNT- and Notch-driven signaling, as well as STAT1/3 signaling and the NRF2-dependent anti-oxidant response. However, little or no effect was observed on TGF-beta/SMAD-signaling (Figure 7).

Thus, it appears that GO treatment somehow targets several different signal transduction pathways in cancer cells, to reduce overall “stemness”.

### GO promotes the differentiation of breast cancer stem cells

**Figure 8:**
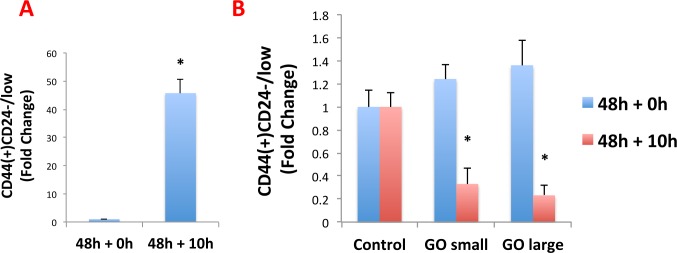
Graphene oxide (GO) promotes the differentiation of breast cancer stem cells MCF7 cells were treated as monolayer cultures with small or big GO (50μg/ml) for 48 hours or left untreated (vehicle alone control). Then, cells were trypsinized and plated on low-attachment plates for 10 hours to induce anoikis and enrich for cancer stem cells. Single cells were then analyzed by FACS to quantitate the CD44(+)CD24-/low population, which represents the cancer stem cells. A. Note that, as expected, the CD44(+)CD24-/low population is greatly enriched after 10 hours in low-attachment conditions (vehicle alone control). B. Interestingly, GO does not reduce the total number of anoikis-resistant cells (data not shown), but rather induces the expression of CD24, thereby significantly reducing the CD44(+)CD24-/low population. This suggests that GO inhibits mammosphere formation by promoting the differentiation of breast cancer stem cells. An * indicates p < 0.05 (Student's t-test).

To further validate the idea GO was reducing stemness in MCF7-derived CSCs, we used a series of well-established breast cancer stem cell markers (CD44 and CD24), and quantitatively analyzed their expression by FACS analysis. The results of these studies are presented in Figure 8.

Briefly, MCF7 cells were treated as monolayer cultures with GO (50μg/ml) for 48 hours or left untreated (vehicle alone control). Then, cells were trypsinized and plated on low-attachment plates for 10 hours to induce anoikis and enrich for CSCs. Single cells were then analysed by FACS to quantitate the CD44(+)CD24-/low population, which represents the breast CSCs. As predicted, the CD44(+)CD24-/low population is greatly enriched after 10 hours in low-attachment conditions (vehicle alone control) (Figure 8A).

Interestingly, GO does not reduce the total number of anoikis-resistant cells (data not shown), but rather induces the expression of CD24, thereby significantly reducing the CD44(+)CD24-/low population (Figure 8B). This suggests that GO inhibits mammosphere formation by promoting the differentiation of breast cancer stem cells, supporting our results from the analysis of multiple signal transduction pathways.

## DISCUSSION

Here, we show that treatment with GO is sufficient to inhibit tumor-sphere formation in six independent cancer cell lines, across multiple tumor types (breast, ovarian, prostate, lung, and pancreatic cancer, as well glioblastoma (brain cancer)). These results suggest that GO specifically targets a global phenotypic property of CSCs that is highly conserved in multiple tumor types. Moreover, using MCF7 cells expressing a panel of luciferase reporters, we observed that GO treatment was indeed sufficient to inhibit a number of different signal transduction pathways, including WNT, Notch, STAT1/3 and NRF-2, but did not effect TGB-beta/SMAD signaling. Finally, using a panel of specific well-established breast CSC markers (namely CD44 and CD24), we show that GO appears to reduce the number of CSCs by inducing their differentiation, as they now begin to express CD24. Thus, GO may reduce the number of bonafide CSCs that are capable of forming tumor-spheres, by inducing their differentiation and inhibiting their proliferation. However, additional mechanistic studies are clearly warranted.

Importantly, our preliminary results indicate that GO treatment does not significantly affect oxidative mitochondrial metabolism (OXPHOS) in this context (data not shown), suggesting that GO does not target mitochondria. This is in contrast to our previous studies where a number of mitochondrially-targeted FDA-approved antibiotics effectively eradiated CSCs [24]. Thus, GO and mitochondrially-targeted antibiotics appear to work differently, via separate and distinct molecular mechanism(s).

**Figure 9:**
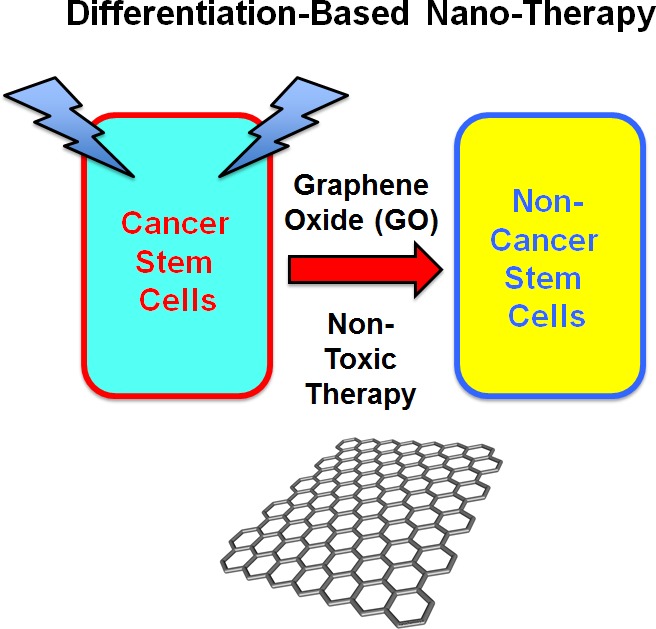
Graphene oxide (GO): Targeting cancer stem cells with differentiation-based nano-therapy Our current mechanistic studies suggest that GO could directly be used as a therapeutic for targeting CSCs, because of its ability to induce differentiation. In this context, we might envision that GO could used to clear residual CSCs, with the aim of preventing tumor recurrence and distant metastasis, thereby providing a practical means for achieving “differentiation-based nano-therapy”.

Also, since b-GO flakes are 5-to-20μm in size, they must be exerting their effects at the cell surface, as they are too large to be internalized within cells and are actually larger than a single cell. This is consistent with our findings that GO-treatment dampens the activation of several stem cell associated signal transduction pathways, which are initiated at the cell surface. This could then mechanistically induce CSC differentiation, which we observed experimentally (summarized in Figure 9).

Previous studies have shown that GO (or its related derivatives) can inhibit “bulk” cancer cell migration or prevent tumor growth in xenograft models [25-28]. However, none of these studies connected GO treatment to the CSC phenotype or indicated that it could be used for “differentiation” therapy.

Interestingly, several studies have shown that GO is non-toxic for normal stem cells, and indeed GO promotes their differentiation. More specifically, it was demonstrated that culturing normal pluripotent stem cells on GO as a substrate induces their terminal differentiation towards multiple cell lineages, including neurons, chondrocytes and adipocytes [29-33]. These properties are currently being actively exploited for tissue engineering and regenerative medicine, by using GO as a scaffold for bone reconstruction and neural regeneration.

Our new mechanistic studies suggest that GO could directly be used as a therapeutic for targeting CSCs, possibly as a differentiation agent. In this context, we envision that GO could be delivered i.v. or p.o., as a new anti-cancer therapeutic, depending on the location of the tumor. Alternatively, GO flakes could also be used as a lavage solution during surgery, to clear the tumor excision site or the peritoneal cavity (as in ovarian or other peritoneal cancers) of residual CSCs, with the aim of preventing tumor recurrence and distant metastasis, via differentiation-based nano-therapy (Figure 9).

## MATERIALS & METHODS

### Materials

Cancer cell lines were purchased from the ATCC or other commercially available sources. Gibco-brand cell culture media (DMEM/F12) was purchased from Life Technologies.

### Graphene oxide

Graphene oxide was prepared by using the Hummers method with modifications [34, 35]. The individual graphite oxide flakes contain carboxyl groups mainly at the edges, and epoxide, hydroxide and ketone groups mainly on the basal plane. The C to O ratio is usually slightly lower or slightly higher than 1 as determined by X-ray photoemission spectroscopy. The graphene oxide flakes of different sizes were separated by centrifuging graphene oxide suspensions at various rpm and collecting different phases of the suspension. The AFM characterization of graphene oxide flakes was performed on a Bruker Dimension FastScan AFM system by using taping mode. The substrates were prepared by spin-casting the suspension on a Si/SiO2 substrate to yield monolayer film, followed by AFM imaging. Concentrations were obtained from UV-Vis spectra, which were recorded in 10 mm path length quartz cells using a PerkinElmer Lambda – 1050 UV-Vis-NIR spectrometer. The dispersions were diluted to give the absorption intensity lower than 1.

### Tumor-sphere culture

A single cell suspension was prepared using enzymatic (1x Trypsin-EDTA, Sigma Aldrich, #T3924), and manual disaggregation (25 gauge needle) to create a single cell suspension. Cells were plated at a density of 500 cells/cm^2^ in mammosphere medium (DMEM-F12/B27/20ng/ml EGF/PenStrep) in non-adherent conditions, in culture dishes coated with (2-hydroxyethylmethacrylate) (poly-HEMA, Sigma, #P3932) [12]. Cells were grown for 5 days and maintained in a humidified incubator at 37°C at an atmospheric pressure in 5% (v/v) carbon dioxide/air. After 5 days for culture, spheres >50 μm were counted using an eye graticule, and the percentage of tumor-sphere formation was normalized to 100% for vehicle alone control (1 = 100 % TSF) [12]. All experiments were performed in triplicate, three times independently, such that each data point represents the average of 9 replicates.

### Evaluation of CSC signalling pathways

The Cignal Lenti reporter assay (luc) system (Qiagen) was chosen for monitoring the activity of several signal transduction pathways in MCF7 cells. The responsive luciferase constructs encode the firefly luciferase reporter gene under the control of a minimal (m)CMV promoter and tandem repeats of response elements for each pathway. The following constructs were used: TCF/LEF(luc) for Wnt signal transduction (CLS-018L); STAT3(luc) for transcriptional activity of STAT3 (CLS-6028L); RBP-Jk(luc) for Notch-induced signaling (CLS-014L); ARE(luc) for Nrf2- and Nrf1-mediated antioxidant response (CLS-2020L); GAS(luc) for Interferon gamma-induced Stat1-signal transduction (CLS-009L); SMAD(luc) for TGFβ-induced signal transduction (CLS-017L). Briefly, 1 × 10^5^ MCF7-GFP cells were seeded in 12-well plates. Once cells were attached, the viral particles were diluted 1:10 in complete culture media containing polybrene (sc-134220, Santa Cruz), and added to the cells. Puromycin treatment (P9620, Sigma) was started 48 hours later in order to select stably infected cells.

### Luciferase assays

The Luciferase Assay System (E1501, Promega Kit) was used on all luciferase reporter MCF7 cells treated with GO. Briefly, 6 × 10^3^ MCF7-GFP cells were seeded in black-walled 96-well plates and then were treated with GO (50 μg/ml). As controls, vehicle-treated cells were run in parallel. Four replicates were used for each condition. After 48 hours of treatment, luciferase assays were performed according to the manufacturer's instructions. Light signal was acquired for 2 minutes in photons/second in the Xenogen VivoVision IVIS Lumina (Caliper Life Sciences), and the results were analysed using Living Image 3.2 sofware (Caliper Life Sciences). Luminescence was normalized using SRB (to determine total cellular protein), as a measure of MCF7 cell viability.

### Anoikis and CSC differentiation analysis

Following GO treatment, the CSC population was enriched by seeding on low-attachment plates. Under these conditions, the non-CSCs undergo anoikis (a form of apoptosis induced by lack of proper attachment) and CSCs are believed to survive. The expression of differentiation markers by the surviving “CSC fraction” was analyzed by FACS analysis. Briefly, 1 × 10^4^ MCF7 cells were treated with GO (50μg/ml) for 48h in 6-well plates, grown as a monolayer. Then, the monolayer cells were trypsinized and seeded in low-attachment plates in mammosphere media. After 10h under low-attachment conditions, MCF7 cells were spun down and incubated with CD24 (IOTest CD24-PE, Beckman Coulter) and CD44 (APC mouse Anti-Human CD44, BD Pharmingen cat.559942) antibodies for 15 minutes on ice. Cells were rinsed twice and incubated with LIVE/DEAD dye (Fixable Dead Violet reactive dye; Invitrogen) for 10 minutes. Samples were then analyzed by FACS (Fortessa, BD Bioscence). Only the live population, as identified by the LIVE/DEAD dye staining, was analyzed for CD24/CD44 expression. Data were analysed using FlowJo software. Virtually identical results were also obtained using 7-AAD (7-Aminoactinomycin D; Life Technologies) to distinguish between the live and dead populations of cells (cell viability), during anoikis.
